# Supplementary material for: Olfactory connectivity mediates sleep-dependent food choices in humans
Source: eLife. 2019 Oct 8;8:e49053. doi: 10.7554/eLife.49053 (PMC6783266; doi:10.7554/eLife.49053)
Supplement: Supplementary file 1. — Means ± SEM of time in bed (TIB), total sleep time (TST), wake after sleep onset (WASO), and sleep efficiency (SE) in the DS and NDS session for the week of sleep stabilization (average across seven nights) and the night of sleep manipulation. P-values from paired t-tests of the difference between NDS and DS sessions. [file elife-49053-supp1.docx]

**Supplementary File 1. Sleep monitoring (actigraphy) data.**

|  | **Sleep stabilization (7-night average)** | | | **Sleep manipulation** | | |
| --- | --- | --- | --- | --- | --- | --- |
|  | **DS** | **NDS** | **P-value** | **DS** | **NDS** | **P-value** |
| TIB | 477.88±8.29 | 476.20±9.36 | 0.463 | 261.56±11.03 | 482.68±5.40 | 1.68x10^-13^ |
| TST | 405.40±7.71 | 398.96±9.40 | 0.174 | 227.20±9.83 | 405.44±10.37 | 6.89x10^-16^ |
| WASO | 71.95±5.03 | 72.92±5.23 | 0.899 | 33.52±4.82 | 75.68±8.51 | 7.62x10^-05^ |
| SE | 84.71±0.99 | 83.68±1.04 | 0.419 | 87.03±1.49 | 83.93±1.79 | 0.108 |
