# Supplementary material for: Olfactory connectivity mediates sleep-dependent food choices in humans
Source: eLife. 2019 Oct 8;8:e49053. doi: 10.7554/eLife.49053 (PMC6783266; doi:10.7554/eLife.49053)
Supplement: Supplementary file 2. — Means ± SEM of ratings for pleasantness, intensity, quality (sweet vs savory), and edibility for the six food and two non-food odors used in this study. For each subject, four food and two non-food odors were presented. Numbers in parenthesis indicate the number of subjects for which a given odor was selected. F- and P-values from one-way ANOVAs across odors. [file elife-49053-supp2.docx]

**Supplementary File 2. Ratings of odor stimuli during screening session.**

|  | **Pleasantness** | **Intensity** | **Sweet vs. savory** | **Edibility** |
| --- | --- | --- | --- | --- |
| Caramel (15) | 6.66±0.15 | 5.78±0.19 | 7.65±0.56 | 8.60±0.29 |
| Cinnamon bun (20) | 6.72±0.13 | 5.98±0.15 | 8.45±0.33 | 8.57±0.25 |
| Ginger cookies (15) | 6.58±0.21 | 6.09±0.15 | 6.98±0.46 | 8.87±0.26 |
| Potato chip (13) | 6.40±0.16 | 5.73±0.17 | -8.60±0.29 | 8.51±0.28 |
| Pot roast (22) | 6.83±0.22 | 6.01±0.19 | -8.49±0.35 | 8.94±0.27 |
| Garlic bread (15) | 6.66±0.22 | 6.81±0.13 | -8.32±0.45 | 8.62±0.23 |
| Fir (25) | 5.55±0.33 | 6.49±0.18 | 0.59±0.69 | 2.22±0.36 |
| Celery seed (25) | 5.07±0.30 | 6.33±0.20 | 1.67±0.79 | 4.21±0.51 |
|  |  |  |  |  |
| One-way ANOVA F,P | 9.56, 5.36x10^−10^ | 3.5, 0.0017 | 955.98, 2.14x10^−62^ | 69.83, 1.67x10^−42^ |
